# From Lock‐and‐Key to Velcro: Glycan‐Dependent T Cell Recruiter Redefines Cancer Cell Targeting With Density‐Dependent Recognition

**DOI:** 10.1002/mco2.70640

**Published:** 2026-02-11

**Authors:** Pengcheng Wei, Aiping Tong, Lin Zhao

**Affiliations:** ^1^ Department of Paediatrics Key Laboratory of Birth Defects and Related Diseases of Women and Children West China Second University Hospital, State Key Laboratory of Biotherapy and Collaborative Innovation Center of Biotherapy, Sichuan University Chengdu China; ^2^ State Key Laboratory of Biotherapy and Cancer Center, West China Hospital West China Medical School, Sichuan University Chengdu China

1

In a recent landmark study published in *Cell*, Zhou et al. uncovered a pivotal breakthrough in cancer immunotherapy: the “glycan‐dependent T cell recruiter” (GlyTR). GlyTR achieves its innovative targeting through a “Velcro‐like” multivalent recognition domain that enables density‐dependent recognition of tumor‐associated carbohydrate antigens (TACAs) on the surface of cancer cells. This innovation offers a novel, safe, and effective solution for pan‐cancer immunotherapy [1].

Conventional immunotherapies, such as bispecific antibodies and CAR‐T cell therapies, rely on a high‐affinity “lock‐and‐key” binding mode to target proteins specifically expressed on tumor cells. However, they often fail to avoid cross‐reactivity with normal tissues that express low levels of the same antigen, leading to off‐target toxicity and severe side effects. Meanwhile, although the abundant glycosylation modifications on tumor cell surfaces are considered promising new targets, their structural complexity and lack of major histocompatibility complex (MHC)‐mediated presentation have long hindered effective antibody‐based targeting [2]. The GlyTR molecules developed by Zhou et al. overcome this longstanding challenge: GlyTR1 utilizes the plant lectin L‐PHA (*Phaseolus vulgaris*, leucoagglutinin) to target β1,6‐GlcNAc‐branched N‐glycans, while GlyTR2 employs human CD301 lectin to simultaneously recognize five TACAs, such as Tn, sialyl‐Tn, GD2, GM2, and LacDiNAc, offering a novel pathway for immune targeting of carbohydrate antigens [1].

While the affinity of GlyTR proteins for TACAs is 3–5 orders of magnitude lower than that of traditional antibodies for protein antigens, GlyTR proteins achieve “density‐dependent” targeting through their multivalent glycan‐recognition domains. This enables the formation of stable complexes specifically on cancer cells with high TACA density [1]. Notably, GlyTR proteins exhibited picomolar‐level cytotoxicity against both solid tumors and hematological malignancies in in vitro experiments using primary cells and organoids. In humanized mouse models, they also induced significant tumor regression—with no observed “on‐target, off‐tumor” toxicity [1]. Furthermore, GlyTR1 demonstrates an inherent immune checkpoint inhibitory function by disrupting the immunosuppressive galectin lattice formed by β1,6‐branched glycans. This mechanism reverses multiple immunosuppressive effects within the tumor microenvironment (TME).

Compared with traditional high‐affinity antibody targeting technologies, GlyTR exhibits fundamental differences in targeting mechanism, antigen selection, and toxicity profile. Currently, most anti‐glycan antibodies show low efficiency in recognizing TACAs; to date, no high‐affinity antibodies against β1,6‐branched glycans have been successfully developed. Although high‐affinity antibodies against glycopeptides (e.g., Tn‐MUC1) have been used to generate potent CAR‐T cells, these antibodies fail to elicit responses in approximately 50% of Tn‐positive cancers [3]. While bispecific T‐cell engagers (BiTEs) and CAR‐T cells based on high‐affinity antibodies have achieved remarkable success in hematological malignancies, their application is limited by on‐target toxicity toward normal tissues that express low levels of the target antigen (Figure 1A) [3]. For instance, anti‐CD19 CAR‐T therapy causes B‐cell aplasia, and anti‐HER2 antibodies carry a risk of cardiotoxicity [3]. In contrast, GlyTR technology not only overcomes the challenge of TACA targeting but also converts “low affinity” into “high avidity” through multivalent binding, enabling the selective killing of targeted cancer cells [1]. Furthermore, this mechanism allows the technology to achieve selective tumor killing across a broad spectrum of cancers while largely reducing on‐target off‐tumor effects on normal tissues—capitalizing on the fact that TACAs are overexpressed in approximately 90% of cancers [3]. The data indicated that a 6–8‐fold higher surface TACA density on cancer cells compared to normal tissues was sufficient for GlyTR to achieve specific tumor killing [1].

**FIGURE 1:**
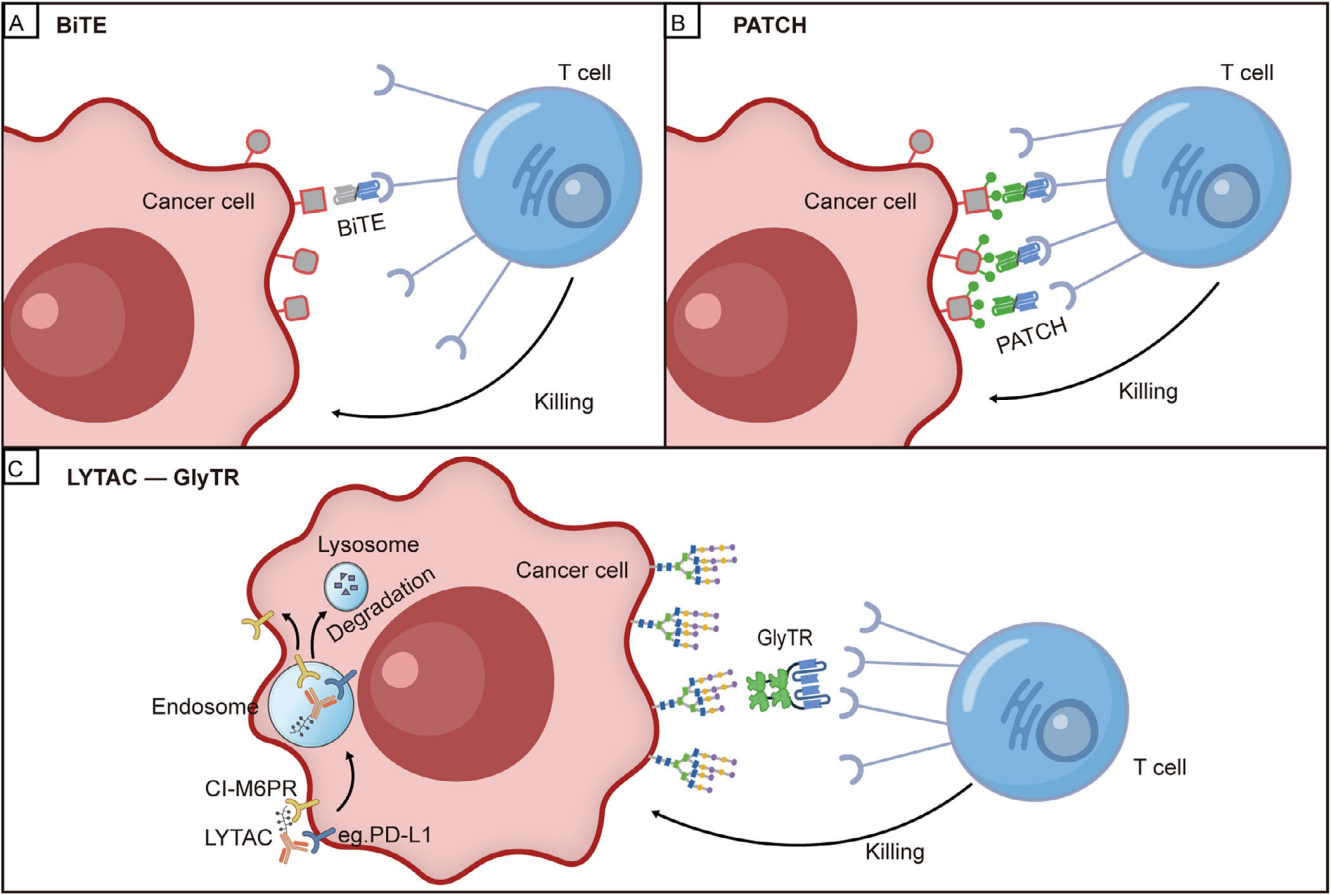
Comparison of mechanisms of action between traditional and novel T‐cell‐recruiting immunotherapies. (A) Mechanism of action of traditional bispecific T‐cell engagers (BiTEs). BiTEs utilize one end of a high‐affinity antibody to target specific protein antigens on the tumor surface, while the other end binds to the CD3 molecule expressed on T cells. This dual‐binding interaction serves to activate T cells and subsequently mediate the killing of tumor cells. (B) The potential of localized antigen amplification (PATCH) technology is an artificial strategy for constructing high‐density antigens [4]. First, through proximity amplification reactions, it synthesizes a large number of artificial haptens (e.g., FITC) in situ on the surface of tumor cells, thereby creating a high‐density antigen microenvironment. Subsequently, bispecific adapters capable of simultaneously recognizing both FITC and CD3 are recruited to this site, bridging T cells with tumor cells and ultimately triggering T cell‐mediated killing. This technology shares a similarity with GlyTR in that both rely on high antigen density to drive T‐cell recruitment; however, GlyTR directly exploits naturally overexpressed carbohydrate antigens on tumors, whereas PATCH artificially constructs antigen density using chemical tools. (C) The left panel illustrates Lysosome‐Targeting Chimeras (LYTACs), which are bifunctional molecules [5]. One end binds to cell‐surface proteins (such as EGFR or PD‐L1), while the other end binds to lysosome‐targeting receptors, thereby guiding the internalization and transport of the target protein to the lysosome for degradation. This results in downregulation of protein expression rather than direct cell killing. The right panel illustrates the glycan‐dependent T cell recruiter (GlyTR). GlyTR is a novel immunotherapy strategy that relies on “density‐dependent” recognition of naturally occurring, high‐density glycosylated antigens on tumor cell surfaces via multivalent lectin domains, leading to direct recruitment and activation of T cells for killing [1]. It utilizes multivalent lectin domains for high‐avidity, “Velcro‐like” binding to densely expressed tumor‐associated carbohydrate antigens, while simultaneously activating T cells through its anti‐CD3 scFv domain, thereby directly inducing T cell‐mediated tumor killing.

Similarly, addressing the issue of insufficient density or poor specificity of target antigens on tumor surfaces, the “potential of localized antigen amplification” (PATCH) technology was recently reported by Li et al. (Figure 1B) [4]. uses catalytic proximity‐labeling reactions to construct high‐density artificial antigen clusters in situ on target cell surfaces, thereby bypassing limitations posed by inadequate or heterogeneous expression of natural antigens [2]. In contrast, GlyTR does not create new antigens but instead efficiently recognizes and exploits naturally overexpressed TACAs on tumor cell surfaces through its multivalent “Velcro‐like” structure, achieving specific and potent killing. Thus, the two technologies have distinct application scenarios and potential combinatorial logic.

GlyTR technology is a new targeted therapy strategy that binds to tumor cell membrane receptors. They use different mechanisms and have different target scopes. When compared further with the newly developing Lysosome‐Targeting Chimeras (LYTAC) technology, it shows potential for complementarity, as seen in Figure 1C. LYTACs use a bispecific design that connects antibodies to lysosomal targeting receptors such as CI‐M6PR [2]. They make use of the endosome‐lysosome trafficking pathway to guide surface membrane proteins, including EGFR and PD‐L1, to the lysosome for degradation [2, 5]. But LYTACs depend on antibodies recognizing protein epitopes and cannot target carbohydrate antigens. Their mechanism affects tumor growth indirectly by lowering the levels of target proteins instead of directly activating immune effector cells [2].

By contrast, GlyTR proteins directly recruit and activate T cells for cytotoxicity, targeting densely expressed TACAs on cancer cells. This creates complementary approaches in terms of targeting level (carbohydrates vs. proteins) and mode of action (immune activation vs. protein degradation) (Figure 1C). Despite their differing mechanisms, GlyTR and LYTAC technologies show promising synergy in combination strategies. LYTAC‐mediated degradation of immunosuppressive proteins (e.g., PD‐L1, CTLA‐4) can alleviate TME suppression, thereby enhancing GlyTR‐driven T cell cytotoxicity [2]. Conversely, GlyTR‐mediated elimination of tumor subsets with high TACA expression may create a more accessible microenvironment for LYTACs. Furthermore, in tumors overexpressing β1,6‐branched glycans, GlyTR1 can block glycan‐mediated immunosuppression, while LYTACs can degrade relevant growth factor receptors (e.g., PD‐L1), establishing dual‐pathway inhibition [2]. Preclinical evidence indicates that combining anti‐PD‐L1 LYTACs with anti‐CD3 bispecific antibodies increases tumor killing efficacy by more than threefold, providing a theoretical basis for the GlyTR‐LYTAC combination strategy [2, 5].

Despite its substantial innovative potential, GlyTR technology still faces several challenges in clinical translation. First, its efficacy is strictly dependent on the expression density of TACAs, which may limit its effectiveness in early‐stage tumors or tumors with high heterogeneity. Second, long‐term safety requires further verification—particularly in normal tissues with high baseline TACA expression (e.g., intestinal brush border, glomeruli). Although no toxicity was observed in animal models, the risk of long‐term exposure in humans remains unclear [1]. In addition, TACA downregulation represents a potential drug resistance mechanism; however, reduced expression of β1,6 branches and Tn may impair the metastatic capacity of tumors, which could yield therapeutic benefit [3]. Furthermore, within the actual TME, likely that subtypes of cancer cells with both high and low TACA expression coexist. The in vitro and in vivo models used in this study employed genetically edited or selected cell lines with uniform TACA expression levels, which failed to replicate such heterogeneity [1]. Consequently, it remains unclear whether GlyTR therapy would selectively eliminate the TACA‐high subclones while sparing the low‐expression ones, potentially leading to the emergence of drug‐resistant tumors dominated by TACA‐low subtypes.

Cancer‐targeted therapy's paradigm is moving toward a convergent model. It strategically arranges GlyTR‐driven immune activation together with LYTAC‐mediated degradation. This synergy seeks to create an all‐around therapeutic network. In the meantime, tapping into tumor glycomic and proteomic data is emerging as the foundational precision guidance system for the next generation of immunotherapies.

## Author Contributions

P.W., A.T., and L.Z. wrote the paper. All authors have read and approved the article.

## Funding

This work is supported by the National Natural Science Foundation of China (32570933, 32300578, and 32471551).

## Ethics Statement

The authors have nothing to report.

## Conflicts of Interest

The authors declare no conflicts of interest.

## Data Availability

The authors have nothing to report.
